# Treatment outcomes of sofosbuvir/velpatasvir/voxilaprevir among NS5A inhibitor‐experienced patients with hepatitis C: Real‐world data from a multicenter Asian registry

**DOI:** 10.1111/jgh.15918

**Published:** 2022-06-30

**Authors:** YJ Wong, R Kumar, R Kumar, J Tan, CH Liu, VW‐K Hui, SS Tan, JH Kao, GL‐H Wong, PH Thurairajah

**Affiliations:** ^1^ Changi General Hospital Changi Singapore; ^2^ Duke‐NUS Medicine Academic Clinical Program, Singhealth Singapore; ^3^ Singapore General Hospital Singapore; ^4^ National Taiwan University Hospital Taipei Taiwan; ^5^ Medical Data Analytics Centre The Chinese University of Hong Kong Hong Kong; ^6^ Selayang Hospital Batu Caves Selangor Malaysia; ^7^ National University Hospital Singapore

*To the Editor,*

Hepatitis C virus (HCV) infection affects over 10 million people in Asia, with widely varying distributions of HCV genotypes across the region and greater heterogeneity than in North America and Europe.^1^, ^2^ While current pan‐genotypic direct‐acting antivirals (DAAs) are highly efficacious, treatment failures still exist, particularly among patients with genotype 3 and decompensated cirrhosis.^3^, ^4^ Sofosbuvir/velpatasvir/voxilaprevir (SOF/VEL/VOX) is indicated as rescue therapy in the event of DAA failure^5^—based on landmark phase 3 trials demonstrating a high sustained virological response rate at 12 weeks (SVR12) of 96–98% in DAA‐experienced HCV patients.^6^

However, data on the efficacy and safety of SOF/VEL/VOX among genetically diverse Asian HCV patients remain scarce. Only 3% of included patients in the existing phase 3 trials were Asian.^6^ Furthermore, data on treatment outcomes with SOF/VEL/VOX as retreatment following SOF/VEL were limited and conflicting.^7^, ^8^, ^9^ To address these gaps, we sought to determine the real‐world efficacy and safety of SOF/VEL/VOX as rescue therapy among NS5A‐experienced HCV patients in Asia.

This was a multicenter, retrospective study involving NS5A inhibitor‐experienced HCV patients from Singapore, Taiwan, Hong Kong, and Malaysia. Eligible subjects were identified through clinic or investigator databases between January 2019 and December 2021. Patients' records were individually reviewed, and data were extracted using a standardized form by a local study team. The primary outcome measure was SVR12, and the secondary outcome was the frequency of adverse events (AEs). The study was conducted in accordance with the Declaration of Helsinki. Consent waiver was obtained from local Institutional Review Boards.

Patients were treated with fixed‐dose SOF 400 mg, VEL 100 mg, and VOX 100 mg (Vosevi®, Gilead Sciences, Foster City, CA, USA) taken orally once daily for 12 weeks, with or without ribavirin, and monitored as per clinical practice guidelines.^4^, ^10^ SVR12 was determined using a real‐time polymerase chain reaction assay (Roche COBAS AmpliPrep/TaqMan version 2.0, Roche Molecular System, NJ, USA) with a minimal detection limit of 12 IU/mL. The per‐protocol (PP) analysis included all patients treated with SOF/VEL/VOX. The modified intention‐to‐treat (mITT) analysis included those who completed 12 weeks of SOF/VEL/VOX with available SVR12 results, while the intention‐to‐treat analysis included all subjects started on SOV/VEL/VOX, AEs were recorded throughout treatment and follow up by SVR12. Standard statistical analyses were performed using SPSS version 23.0, with a *P*‐value <0.05 considered statistically significant.

A total of 25 patients were included from six institutions (Table 1). The cohort had a mean age of 55 ± 9 years with male predominance (84.0%). More than half had genotype 3 HCV infection (56.0%) and liver cirrhosis (64.0%). Prior DAA regimens were: SOF/VEL (*n* = 20, 80.0%); SOF/ledipasvir (*n* = 2, 8.0%); SOF/daclatasvir (*n* = 1, 4.0%); glecaprevir/pibrentasvir (*n* = 1, 4.0%); or daclatasvir/asunaprevir (*n* = 1, 4.0%)

**Table 1:** Baseline characteristics of study subjects

Characteristics	Total (*N* = 25)
Age, years	55 (9)
Male	21 (84.0)
Race	
Chinese	10 (40.0)
Malay	9 (36.0)
Indian	4 (16.0)
Other	2 (8.0)
Genotype	
1	2 (8.0)
2	2 (8.0)
3	14 (56.0)
6	2 (8.0)
Indeterminate	5 (20.0)
IVDU	14 (56.0)
HCV RNA, log_10_ IU/mL	5.5 (1.3)
Fibrosis stage	
F1	6 (24.0)
F2	2 (8.0)
F3	1 (4.0)
F4/cirrhosis	16 (64.0)
HBsAg	1 (4.0)
HIV	1 (4.0)
Active HCC	3 (12.0)
Prior DAA therapy	
Sofosbuvir/Velpatasvir	20 (80.0)
Sofosbuvir/Ledipasvir	2 (8.0)
Sofosbuvir/Daclatasvir	1 (4.0)
Glecaprevir/Pibrentasvir	1 (4.0)
Daclatasvir/Asunaprevir	1 (4.0)

All data are *n* (%) or mean (standard deviation). Per‐protocol dataset. DAA, direct‐acting antiviral; HBsAg, hepatitis B surface antigen; HCC, hepatocellular carcinoma; HCV, hepatitis C virus; HIV, human immunodeficiency virus; IVDU, intravenous drug user.

The overall SVR12 was 95.7% (*n* = 22/23) and 88.0% (*n* = 22/25) in the mITT and PP analyses, respectively. SVR12 rates were comparable in a subgroup analysis was performed based on HCV genotype (Genotype‐3: 91.7% *vs* 100.0%; *P* = 0.688) (Fig. 1). Successful treatment resulted in significant improvements in liver biochemistry and fibrosis markers such as FIB‐4 (*P* < 0.05) (Table 2).

**Figure 1:**
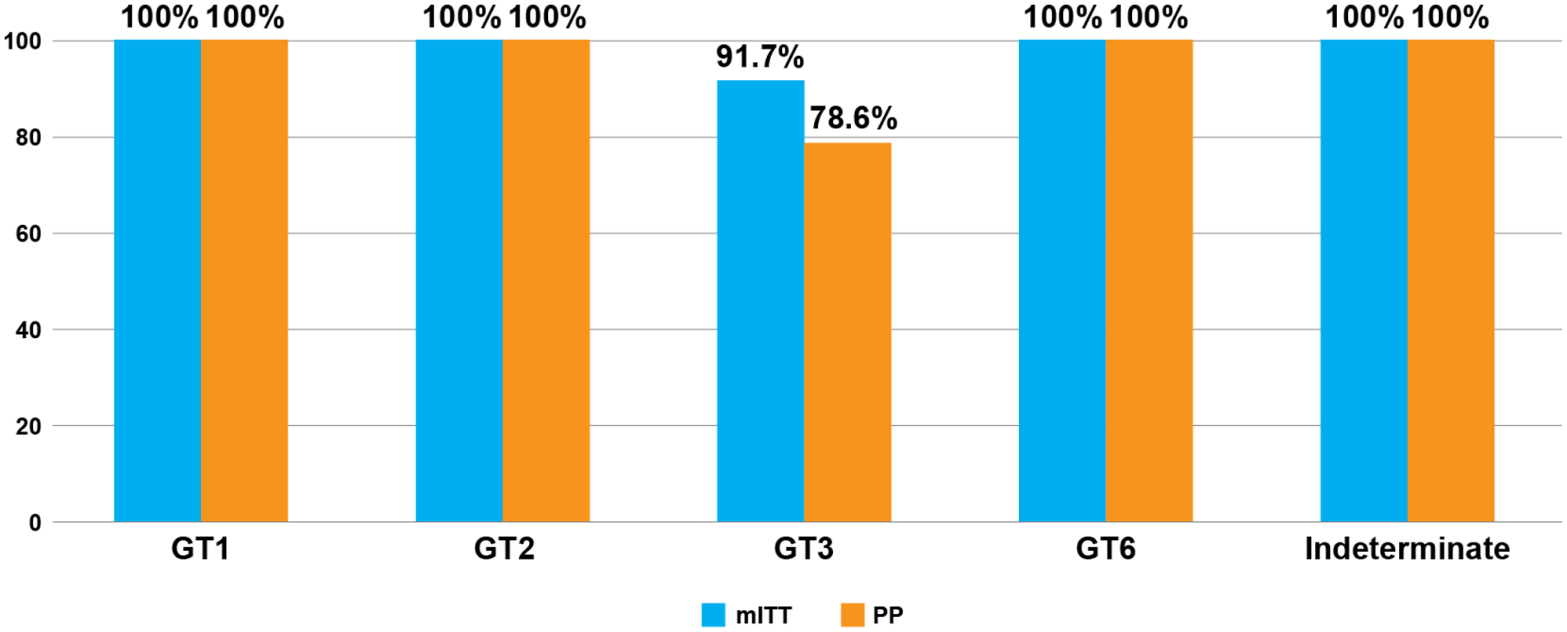
SVR12 rates based on HCV genotypes. GT, genotype; mITT, modified intention‐to‐treat; PP, per protocol; SVR12, sustained virologic response 12 weeks after the end of treatment.

**Table 2:** Laboratory assessments

	Pre‐treatment	Post‐treatment	*P*‐value
ALT, U/L^†^	81 (38–156)	21 (18–42)	**0.002**
AST, U/L^†^	100 (64–148)	30 (24–40)	**<0.001**
AFP, ng/mL^†^	5.3 (2.5–11.7)	3.0 (2.0–7.0)	**0.011**
Albumin, g/L^†^	39 (30–44)	41 (31–44)	0.972
Bilirubin, μmol/L^†^	17 (12–24)	14 (9–25)	**0.005**
INR	1.09 (1.02–1.16)	1.04 (0.98–1.10)	**0.043**
Hemoglobin, g/dL	14.0 (13.0–15.8)	14.9 (13.3–16.0)	0.83
Platelets, 10^3^/mm^3^ ^†^	152 (83–196)	154 (99–193)	0.611
Sodium, mmol/L^†^	139 (136–141)	138 (136–140)	0.826
Creatinine, μmol/L^†^	70 (61–86)	78 (64–88)	0.779
FIB‐4 score	4.58	4.91	**<0.001**

^†^ Mean (range).

Modified intention‐to‐treat dataset (*N* = 25).

AFP, alpha‐fetoprotein; ALT, alanine aminotransferase; AST, aspartate aminotransferase; FIB‐4, fibrosis‐4 index for liver fibrosis; INR, international normalized ratio.

Items in bold indicates *P*‐value < 0.05.

No patients required treatment cessation due to treatment‐related AEs. Two individuals died before completion of SOF/VEL/VOX. The causes of death were hepatocellular carcinoma and septic shock, respectively, which were deemed not related to SOF/VEL/VOX by the investigators. The only subject who experienced virologic failure had genotype 3 compensated cirrhosis with clinically significant portal hypertension. He was currently on transplant wait‐list, with plan for retreatment following liver transplantation.

To the best of our knowledge, this is the largest cohort of Asian HCV patients treated with SOF/VEL/VOX to date, with the majority being “difficult to treat” (78% were genotype 3 with prior SOF/VEL exposure). These findings complement existing efficacy data from the western cohort. Whether SOF/VEL/VOX resulted in a lower SVR12 rates among genotype‐3 HCV patients remained debatable.^7^, ^8^, ^9^, ^11^, ^12^ In this Asian cohort, effectiveness seemed comparable among genotype 3 HCV patients. It is also reassuring that the effectiveness of SOF/VEL/VOX was preserved followed SOF/VEL failure as most of the earlier data were derived from SOF‐based regimens other than SOF/VEL.^7^, ^8^, ^9^

Despite being a multicenter, multinational study, the present study was limited by its retrospective design and a relatively small sample size, which restricted our ability to perform further subgroup analyses. The small number could be related to the high success rate with pangenotypic DAA among Asian HCV patients, as shown in our earlier study.^3^ The resistant pattern was not available because it was not assessed in routine clinical practice. However, the high efficacy of SOF/VEL/VOX in our cohort suggests that routine resistant testing may not be necessary among NS5A‐experienced Asian HCV patients.

In conclusion, this study demonstrates that SOF/VEL/VOX is an efficacious and safe option among NS5A inhibitor‐experienced Asian HCV patients. Safety data are emerging for protease inhibitor‐containing DAAs in patients with decompensated cirrhosis.^13^, ^14^ Further studies are warranted to investigate the safety of SOF/VEL/VOX in this population.

## Funding

YJW is supported by the Nurturing Clinician Scientist Scheme (NCCS) award by SingHealth Duke‐NUS Academic Medical Centre, Singhealth. Medical writing services and open access charges were funded by Gilead Science. The funding agency had no control over the content or design of the study.

## Ethics approval statement

The study was conducted according to the guidelines of the Declaration of Helsinki.

## Patient consent statement

Consent waiver was obtained from the Institutional Review Board.

## Data Availability

Primary data are available upon reasonable request from the corresponding author.
